# Valve-Sparing Root Replacement With Anomalous Left Circumflex From the Right Coronary Sinus

**DOI:** 10.1016/j.atssr.2025.03.002

**Published:** 2025-03-19

**Authors:** Alexander P. Nissen, Woodrow J. Farrington

**Affiliations:** 1 Division of Cardiothoracic Surgery, Department of Surgery, Emory University School of Medicine, Atlanta, Georgia

## Abstract

Valve-sparing root replacement (VSRR) with the reimplantation technique is ideal for most cases of isolated aortic root aneurysm with a well-functioning valve. VSRR is also increasingly applied across a broader spectrum of root pathology with otherwise sparable aortic valves. Recently, there is increased recognition of coronary artery anomalies, which present unique challenges at the time of VSRR. We present a case description and video of our technique for VSRR in a patient with an anomalous left circumflex from the right coronary sinus, with a separate ostium, and retroaortic course.

Since first described, the reimplantation technique for valve-sparing aortic root replacement (VSRR) has demonstrated excellent durability when well-executed.^1^^,^^2^ Applications have also broadened beyond isolated root aneurysms.^3^^,^^4^ Coronary artery anomalies are uncommon in adults, although diagnosis continues to increase with the application of advanced imaging.^5^

Coronary anomalies represent a spectrum of pathology and may influence the execution of VSRR based on which vessel is anomalous, ostial factors (slit-like vs open orifice, ostial relation to aortic commissures), and the anomalous coronary course, which may be at risk of compression, in close proximity to the primary suture line, or both. We present a case of VSRR in a patient with an anomalous left circumflex (LCx) from a separate ostium in the right coronary sinus and a retroaortic course requiring particular consideration during the deep root dissection and primary suture line.

Our patient is a 63-year-old man with a history of hypertension, prior right upper lobectomy for stage 1 lung cancer, and a known aortic root aneurysm under surveillance. His root had grown since 2021 to a size of 5.5 cm, with asymptomatic mild-to-moderate aortic insufficiency, and preserved left ventricular function. Preoperative computed tomography angiography of the chest confirmed his root aneurysm and also demonstrated an anomalous LCx arising from the right coronary sinus, with a retroaortic course. This was confirmed on preoperative left-sided heart catheterization, also demonstrating a dominant right coronary artery (RCA), with no significant coronary atherosclerosis (Figure 1).

**Figure 1:**
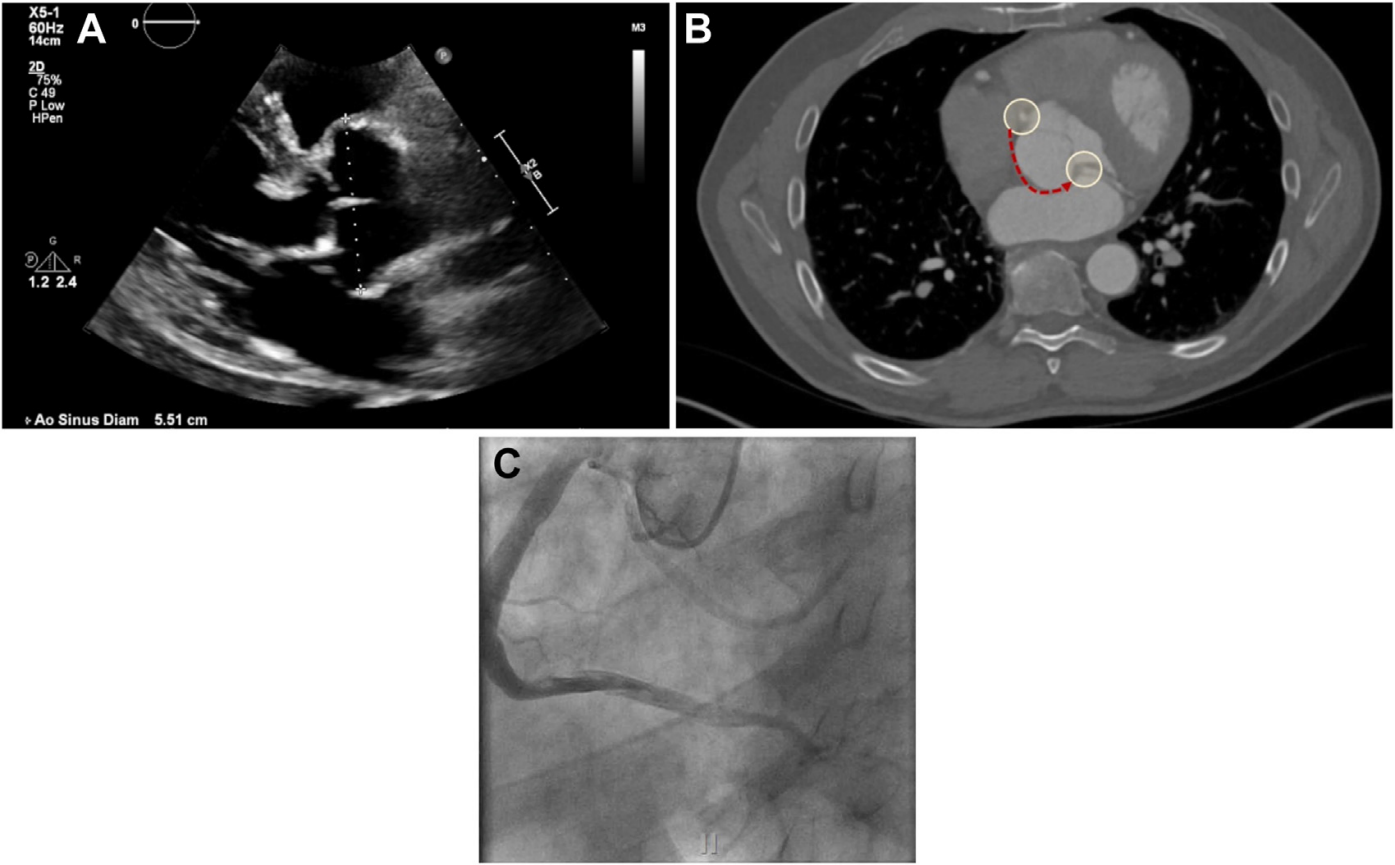
(A) Preoperative transthoracic echocardiogram. (B) Preoperative computed tomography angiography with anomalous left circumflex course highlighted. (C) Preoperative left-sided heart catheterization with opacification of the right coronary artery and the anomalous left circumflex.

## Technique

The Supplemental Video outlines our technique. The heart was exposed through a median sternotomy, and after heparinization, the distal ascending aorta and right atrial appendage were cannulated, with a left ventricular vent through the right superior pulmonary vein. The aorta was cross-clamped and cold blood cardioplegia achieved diastolic arrest. Initial valve inspection demonstrated a trileaflet aortic valve with good cusp mobility and coaptation, confirming candidacy for VSRR.

**Figure 2:**
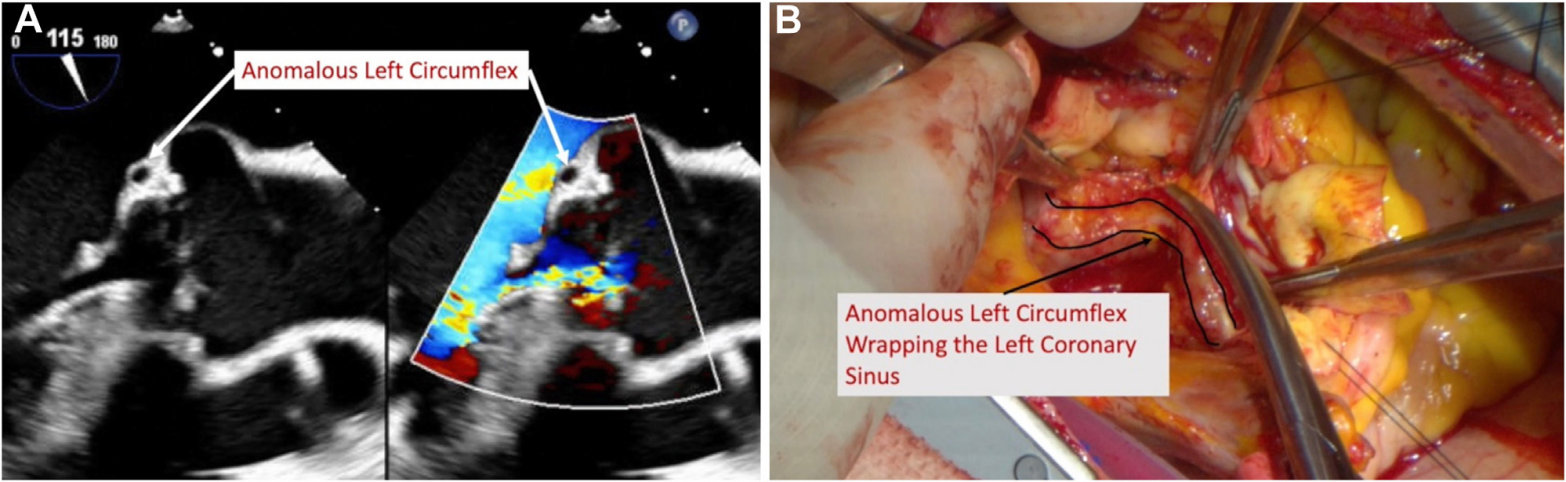
(A) Intraoperative transesophageal echocardiography demonstrates the anomalous left circumflex wrapping the left/noncoronary sinus at/below the level of the annulus. (B) The anomalous left circumflex is dissected along its retroaortic course to accommodate the primary suture line.

The periaortic tissues were dissected down to the level of the annular plane externally, taking care identify and protect the anomalous LCx, particularly because it wraps around the noncoronary sinus in its retroaortic course, which in normal coronary anatomy would be a clear plane down to the level of the annulus (Figure 2). The LCx was dissected sufficiently to allow eventual placement of our subannular sutures without impingement. Additional doses of ostial cardioplegia down the LCx also confirmed no injury or extravasation.

The RCA and anomalous LCx ostia were in close proximity within the right sinus and were harvested as a single button. The left anterior descending button was freed in standard fashion. The remaining sinus tissue was trimmed to leave a 3-mm rim for valve reimplantation. The annulus was sized to a 26-mm Hegar dilator. After sizing, we will typically add 5 mm to the Hegar size to determine the Valsalva graft size of choice, erring larger if between sizes.

A 32-mm Valsalva graft was chosen. A series of five 2-0 Ethibond (Ethicon) pledgeted sutures were placed in the annular plane, 1 at each commissure, 1 at the nadir of the right coronary sinus, and 1 at the nadir of the noncoronary sinus. We elected not to place a subannular stitch at the nadir of the left coronary sinus to avoid any impingement of the aberrant circumflex artery. Our subannular sutures were then passed through the base of the Valsalva graft and lowered into position.

The commissural heights were measured and resuspended within the graft. Each sinus was then reimplanted, beginning at the nadir and running to the commissural apex. After reimplantation, the valve was tested and appeared to coapt at equal heights along the free margins. After the ideal position was confirmed, the left anterior descending button was anastomosed to the Valsalva portion of the graft. Cardioplegia was then delivered down the graft and pressurized, demonstrating valve competency and hemostasis of our suture lines. The ascending portion of the graft was trimmed to length, and our distal anastomosis was performed. Finally, the combined RCA and LCx button was reattached to the Valsalva portion of the graft after determining optimal position with the heart filled.

**Figure 3:**
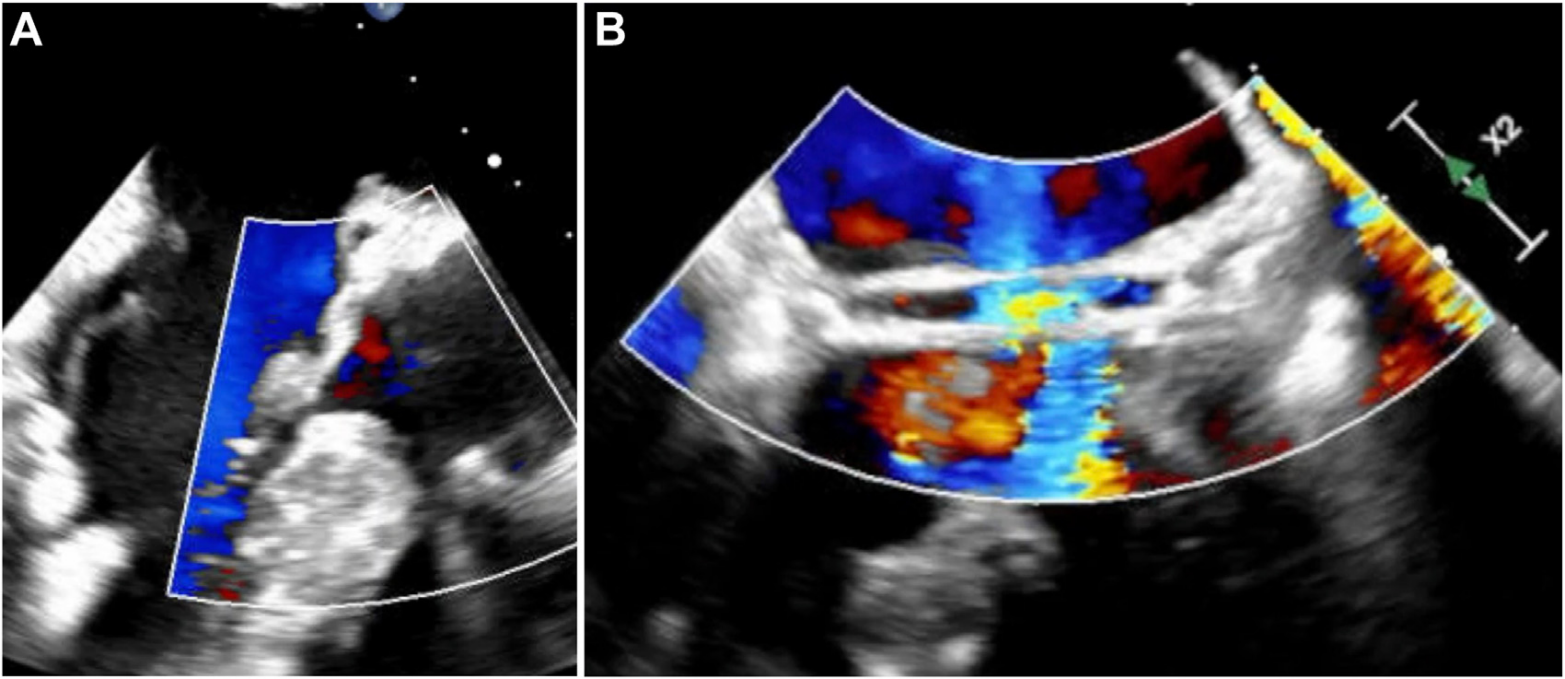
Postrepair transesophageal echocardiography (A) demonstrates preservation of the anomalous left circumflex, with the graft well-seated down to the annular plane, and (B) confirms flow in the left circumflex after valve-sparing root replacement.

After air was removed, the cross-clamp was released and the heart regained sinus rhythm spontaneously. Postbypass transesophageal echocardiography revealed only trace central aortic insufficiency, with flow through each coronary button, good leaflet mobility, and a coaptation height of 7 mm (Figure 3). The patient made an uneventful recovery, was extubated the day of surgery, and discharged home on postoperative day 5.

## Comment

VSRR with the reimplantation technique remains the gold standard for repair of isolated aortic root aneurysms, with several technical considerations critical to success. Coronary anomalies have been previously identified at the time of VSRR, with few successful repairs reported in the literature, whereas the frequency of intraoperative conversion to a Bentall operation will always remain unknown. The presence of anomalous coronaries adds complexity to VSRR, particularly during deep root dissection, primary valve suture line, and coronary button management decisions. Additional consideration must be given to any coronary unroofing, osteoplasty, or reimplantation based on the characteristics of the coronary ostium, any intramural segment, and its course after leaving the aorta.^6^

With careful planning and consideration of these factors, we believe most cases for which VSRR is indicated should be feasible regardless of coronary anatomy. The role for concomitant coronary artery bypass grafting in these cases remains limited.^7^ Theoretically, in cases with an anomalous coronary course that prohibits safe deep root dissection and the primary suture line, one could consider the remodeling technique for VSRR, although this is not our default technique.^8^

Before any VSRR, preoperative computed tomography angiography and left-sided heart catheterization should identify anomalous coronary anatomy. We also demonstrate key findings on preoperative and intraoperative transesophageal echocardiography, which can add important detail regarding the proximity of an anomalous coronary to the aortic annulus and document flow in the artery before and after VSRR.

In conclusion, the presence of anomalous coronaries presents unique challenges at the time of VSRR, which can be overcome by careful planning and technical considerations to avoid pitfalls and preserve excellent outcomes.
